# Antioxidant Potential of Psychotropic Drugs: From Clinical Evidence to In Vitro and In Vivo Assessment and toward a New Challenge for in Silico Molecular Design

**DOI:** 10.3390/antiox9080714

**Published:** 2020-08-06

**Authors:** Giovanni Ribaudo, Marco Bortoli, Chiara Pavan, Giuseppe Zagotto, Laura Orian

**Affiliations:** 1 Dipartimento di Medicina Molecolare e Traslazionale, Università degli Studi di Brescia, Viale Europa 11, 25123 Brescia, Italy; giovanni.ribaudo@unibs.it; 2 Dipartimento di Scienze Chimiche, Università degli Studi di Padova, Via Marzolo 1, 35131 Padova, Italy; marco.bortoli@unipd.it; 3 Dipartimento di Medicina, Università degli Studi di Padova, Via Giustiniani 2, 35128 Padova, Italy; chiara.pavan@unipd.it; 4 Dipartimento di Scienze del Farmaco, Università degli Studi di Padova, Via Marzolo 5, 35131 Padova, Italy; giuseppe.zagotto@unipd.it

**Keywords:** antipsychotic drugs, antidepressants, oxidative stress, radical scavenging, quantum chemistry, molecular design

## Abstract

Due to high oxygen consumption, the brain is particularly vulnerable to oxidative stress, which is considered an important element in the etiopathogenesis of several mental disorders, including schizophrenia, depression and dependencies. Despite the fact that it is not established yet whether oxidative stress is a cause or a consequence of clinic manifestations, the intake of antioxidant supplements in combination with the psychotropic therapy constitutes a valuable solution in patients’ treatment. Anyway, some drugs possess antioxidant capacity themselves and this aspect is discussed in this review, focusing on antipsychotics and antidepressants. In the context of a collection of clinical observations, in vitro and in vivo results are critically reported, often highlighting controversial aspects. Finally, a new challenge is discussed, i.e., the possibility of assessing in silico the antioxidant potential of these drugs, exploiting computational chemistry methodologies and machine learning. Despite the physiological environment being incredibly complex and the detection of meaningful oxidative stress biomarkers being all but an easy task, a rigorous and systematic analysis of the structural and reactivity properties of antioxidant drugs seems to be a promising route to better interpret therapeutic outcomes and provide elements for the rational design of novel drugs.

## 1. Introduction

It is commonly accepted that several clinical conditions are caused or facilitated by oxidative stress, or at least that it contributes to their progression. On the other hand, oxidative stress often results from the disease itself [1]. Moreover, inflammation and oxidative stress appear to be linked in physiological as well as pathological conditions. Consequently, they have been defined as “essential partners” in certain diseases [2].

Even if the brain represents only a small portion of human body weight, neurons of the central nervous system (CNS) use almost 80% of the total energy. The metabolic activity of the brain is high and the subsequent oxygen demand, the unsaturated lipid enrichment and the metabolism of neurotransmitters determine that CNS is highly exposed to the effects of the oxidation–reduction imbalance, mainly involving reactive oxygen (ROS) and nitrogen species (RNS) [3,4].

In this context, Halliwell clearly stated that antioxidants, which may be promising options or supplements for the treatment of several human diseases, can be divided into three main groups, consisting of: (1) endogenous antioxidants, i.e., naturally occurring in the human body, (2) synthetic antioxidants, such as chelating agents contrasting ion-dependent free radical reactions, and (3) drugs which were designed to interfere with other biochemical pathways but may have additional physiological action due to their antioxidant properties [1].

In this review, we focus our attention on the third group. In fact, it has been demonstrated that several drugs, or classes of drugs, already in clinical use, are endowed with antioxidant activity. Several classes of approved drugs have been studied through the years for their antioxidant properties [1,5,6]. Proton pump inhibitors [7], antidiabetics [8], drugs acting on the cardiovascular system [9], antiepileptics [10], and anti-inflammatory agents [11] represent some of the investigated classes, which are also paired by antioxidant natural and semi-synthetic compounds with biological activity [12,13,14,15,16,17,18,19]. Furthermore, besides therapeutic agents, it must be stressed that bioactive components from diet have been recognized among the risk factors or, on the other hand, protective agents possibly influencing oxidative stress and pathogenesis of related diseases [20,21]. More specifically, increased peripheral inflammatory markers, elevated production of ROS, reduced activity of the antioxidant systems and decreased efficiency in repairing mechanisms are associated with mental diseases such as major depressive disorders and schizophrenia, suggesting a direct involvement of oxidative stress in their pathophysiology.

**Figure 1:**
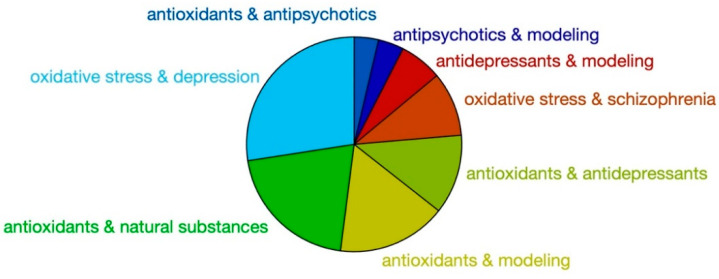
SCOPUS indexed works found with the shown keywords from 1990 (www.scopus.com, site accessed 10 July 2020). Sample size 16,075 documents.

Starting from this point, we looked at the literature of the last 30 years to check how many contributions were present in this research field, using some precise keyword combinations (Figure 1). The results confirm that while the correlation of oxidative stress and depression or schizophrenia has been extensively studied, the antioxidant action of active antipsychotic and antidepressants has been investigated far less.

In this review, we provide a focused perspective on the role of antipsychotic and antidepressant drugs in contrasting oxidative stress in schizophrenia and depression, as to the best of our knowledge no contribution overviewing these classes has been reported to date.

### Oxidative Stress in Schizophrenia and Depression

**Figure 2:**
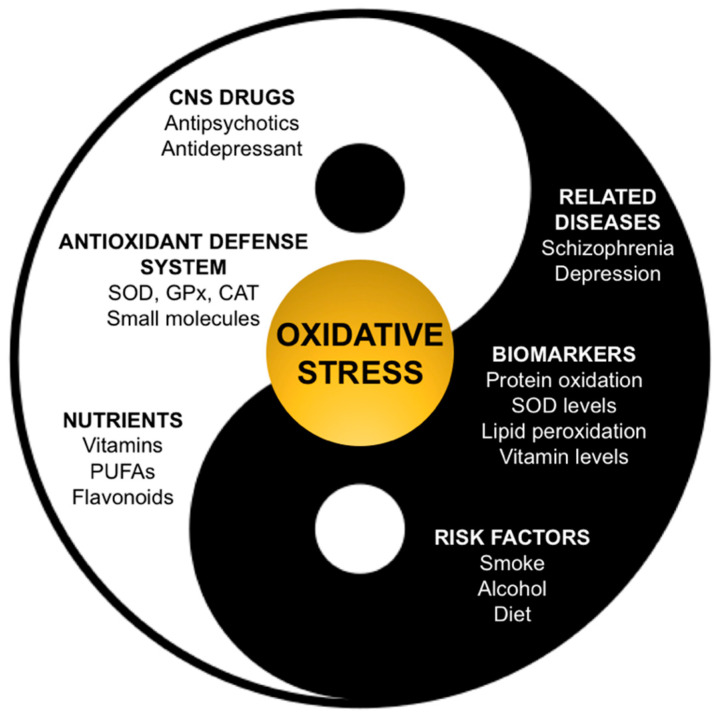
The concept of oxidative stress as a balance between pro-oxidant and antioxidant conditions, which is particularly relevant in the cases of schizophrenia and depression.

Oxidative stress consists of an altered balance between pro-oxidant and antioxidant events. This can result in oxidative damage affecting cell components such as lipids, proteins (including enzymes), carbohydrates and DNA [22]. Most importantly, oxidative stress is a condition which is thought to be involved in the pathogenesis and progression of several psychiatric disorders [23] and, in particular, has been associated with the onset of first-episode psychosis [24,25]. It must be also considered that high cigarette consumption and excessive alcohol intake are also very common in patients with major depression and schizophrenia. It has been postulated that better control of oxidative stress could avoid the starting and a worsening course of a psychiatric illness. In this context, it is well documented that the typical American diet, mostly based on processed foods, is severely deficient in antioxidant compounds and that a highly caloric diet and a high consumption of red meat would increase oxidative stress. As an example, it must be noted that perhydroxyl radical can diffuse in lipids, and it can produce a carbon-centered radical of polyunsaturated lipids [6]. On the other hand, it has been demonstrated that schizophrenic patients have low levels of antioxidant and anti-inflammatory nutrients, such as omega-3 PUFAs, vitamin D, B vitamins, vitamin E and carotenoids, and their supplementation treatment seems to reduce oxidative stress and psychotic symptoms [26]. CNS is particularly subjected and vulnerable to injuries mediated by pro-oxidant reactive species. High oxidative metabolic activity and oxygen consumption, moderate to low concentrations of antioxidant enzymes levels, presence of a high membrane surface area and of a vulnerable neuronal anatomical structure contribute to this susceptibility [22,27]. Moreover, the brain is rich in polyunsaturated fatty acids and does not present cellular turnover, thus it is particularly vulnerable to oxidative injury [6]. It must also be considered that neurotransmitters metabolism itself threatens CNS redox balance: dopamine, epinephrine and norepinephrine degradation can induce the production of H_2_O_2_, and neuronal mitochondria generate superoxide radical (O_2_^•−^). Moreover, the presence of toxic amino acids can promote cell proteolysis [28]. On the other hand, human antioxidant defense system has the physiological role of contrasting these insults. Superoxide dismutase (SOD), glutathione peroxidase (GPx) and catalase (CAT) constitute the enzymatic pool that has the role of blocking adverse chain reactions. Moreover, non-enzymatic components comprehend small molecules such as glutathione (GSH), vitamin E, vitamin C, N-acetyl-cysteine and β-carotene. In principle, pathological changes due to the activity of ROS can be improved through two mechanisms: inactivation of oxiradicals by means of antioxidants introduced with diet (vitamin C, E, β-carotene and ubiquinones), and/or supplementation of esterified fatty acids [29,30,31]. As anticipated, oxidative stress manifests when oxidant insults overcome these defenses, thus resulting in an altered pro-oxidant/antioxidant balance, as in an “out of tune” yin and yang (Figure 2), where food supplements and antioxidant drugs can also play a role. The deficit of one of these components can make cellular membranes vulnerable to ROS damage, causing peroxidation of lipid membranes, alteration of transduction signals that provoke receptor, nuclear and mitochondrial DNA damage and, ultimately, neuronal cell death. In this context, growing evidence supports the involvement of oxidative stress, nitrosative stress and mitochondrial dysfunction in the pathogenic process and declining course of schizophrenia [25,32,33,34].

Regarding the available biochemical markers, CNS oxidative damage can be detected by measuring lipid peroxidation products in cerebrospinal fluid and plasma and by evaluating the reduction of membrane polyunsaturated fatty acids (PUFAs) in the brain and red blood cell membranes [22,35]. PUFAs peroxidation, a possible indicator of disease progression, can be detected through the quantification of lipid peroxides, isoprostanes, aldehydic products and volatile hydrocarbons. Thiobarbituric acid-related substances (TBARS) are considered markers of lipid peroxidation in schizophrenia, although this parameter is characterized by poor specificity [36]. Moreover, detectable oxidative modification of proteins may also be taken into account to trace disease progression. At the cellular level, such damages affect enzyme catalysis, ligand binding, signal transduction and can contribute to secondary damage of other proteins. In this context, bound aldehydes, glycated proteins and homocysteines are generally measured as markers [37]. Oxidative damage could also reflect on nucleic acid status: Nishioka et al. reported that elevated levels of 8-hydroxy-2-deoxyguanosine were detected in schizophrenia patients. Nevertheless, the reliability of this parameter is also debated [22,38]. Besides the markers described above, antioxidant enzymes (SOD, GPx and CAT), vitamin E and vitamin C levels can also be quantified to evaluate the defense system status in schizophrenia, [39] although the significance of positive or negative variations of their concentration (in particular of the enzymatic components) is not completely clear. In fact, while, according to the majority of studies, a decreased antioxidant system would be associated with disease progression, some other reports showed opposite results. In particular, one hypothesis is that antioxidant enzymes levels should be lower at the early stages of psychotic disorders [22,40].

In the context of mental disorders, oxidative stress also plays a role in the onset of depression [41]. Additionally, in this case, the cause–consequence relationship is not very clear. Moreover, the beneficial role of antidepressants on oxidative stress is itself debated, since some therapeutic agents act by interfering with enzymes that are involved in oxidative metabolism. In particular, the activity of monoamine oxidase A and B (MAO-A and MAO-B) leads to the production of hydrogen peroxide as a byproduct of the reaction between the enzymes and their monoamine substrate. Depression is commonly treated with drugs that increase the concentration of biogenic amines, which are substrates for MAOs, and this may cause the generation of neurotoxic aldehydes and increase oxidative stress [42,43,44]. Thus, the metabolism of biogenic amines can itself play a role in the oxidative equilibrium within CNS, and this is especially the case of dopamine (DA), which is fundamental for reward and movement regulation in the brain and is also a precursor in the biosynthesis of other catecholamines such as norepinephrine and epinephrine. Its metabolic transformation is operated by MAO and catechol-O-methyl transferase (COMT) enzymes. However, mitochondrial dysfunction and oxidative stress contribute to the formation of 6-hydroxydopamine (6-OHDA), which is an oxidative stress mediator itself and can contribute to cell death [45]. On the other hand, markers of oxidative stress (vitamins and malondialdehyde levels, TBARS) were found to be higher in depressed patients [46]. 8-hydroxy-2′-deoxyguanosine (8-OHdG), an oxidized derivative of deoxyguanosine, and F2-isoprostanes are two more specific markers, indicating oxidative damage to DNA and lipids, respectively, and were considered in a meta-analysis by Black and colleagues [47,48]. The authors highlighted that 8-OHdG and F2-isoprostanes are increased in subjects with depression (major depressive disorder, bipolar disorder and depressive symptoms). These results point out the association between depression and increased oxidative damage, but the underlying mechanisms should still be clarified, especially in light of the cross-correlation of depression with other behavioral factors that are related to increased exposure to ROS (smoking, alcohol use, overweight condition) [49,50,51].

## 2. Antipsychotic Drugs

**Scheme 1:**
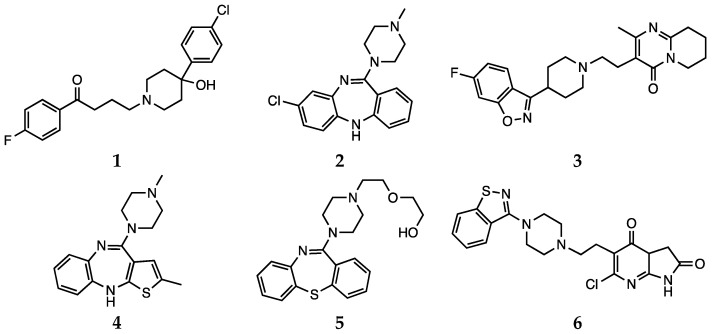
Haloperidol (**1**), clozapine (**2**), risperidone (**3**), olanzapine (**4**), quetiapine (**5**) and ziprasidone (**6**).

Antipsychotic drugs are pharmacological agents that have been introduced over 4 decades ago [52,53]. Currently, treatment options include the use of a single molecule or a combination of antipsychotics. These agents are classified as first-generation, or typical, and second-generation, or atypical. Moreover, a third generation of drugs has been more recently introduced [54]. The mechanism of action of typical antipsychotics (haloperidol, **1**, Scheme 1) consists in blocking dopamine type 2 receptors. Atypical antipsychotics (clozapine, risperidone, olanzapine, quetiapine and ziprasidone, **2**–**6**, Scheme 1), on the other hand, have lower affinity for dopaminergic receptors but also block serotoninergic 5-HT_2A_ receptors [54]. For more details on the molecular mechanisms underlying the activity of antipsychotic drugs and on the pharmacological aspects, the reader is invited to refer to the recent contributions by Aringhieri and colleagues and by Marder and colleagues [55,56]. Although great improvements in the management of schizophrenia were achieved after the introduction of atypical antipsychotic drugs in the early 1990s, it must also be pointed out that their use is associated with some severe adverse effects. Clozapine can cause agranulocytosis, while the use of olanzapine has been connected with hepatotoxicity [57].

On top of the considerations reported above, the effects on oxidative status of CNS of the drugs used to treat schizophrenia has to be clarified, since contrasting reports are present in the literature [58,59]. In fact, growing evidence highlights that CNS oxidative stress status could be influenced by using antipsychotics, although the different behavior of atypical versus typical agents is a controversial issue [60]. 

### 2.1. First Generation (Typical) Antipsychotics

The effects of typical antipsychotics on oxidative stress level is probably the most debated. In fact, evidence suggests that increased lipid peroxidation seems to be associated with the use of these molecules in therapy [61]. Moreover, according to the reports available in the literature, treatment with the typical antipsychotic haloperidol induces a sensible increase in mitochondrial activity in generating toxic reactive species. In particular, the generation of a pyridinium metabolite is thought to be responsible for cytotoxicity, extrapyramidal side effects and cardiac functional disorders [61,62,63]. The antioxidant role of haloperidol was also investigated in a more recent study by Brinholi et al. (discussed below). The compound was not found to be very effective in the in vitro antioxidant tests [53]. Haloperidol was also described to induce lipid peroxidation in schizophrenic patients [64]. The fact that the treatment with such antipsychotics would not lead to unambiguous results is further supported by clinical evidence. Kriisa et al. reported the results of a study conducted considering several indices of oxidative stress in first-episode psychosis patients. The patients were given typical, atypical or mixed medications and oxidative stress markers (total antioxidant capacity, lipid peroxidation and protein oxidation) were measured in blood. First, the authors highlighted the absence of significant differences in such levels between first-episode psychosis patients and the control group. Anyway, the antipsychotic treatment induced two positive effects: a decrease in oxidative status and an amelioration of inflammation. Nevertheless, the authors pointed out that these effects were not observed in long-term chronic schizophrenia patients, who were showing significant high-grade oxidative stress [25].

### 2.2. Second Generation (Atypical) Antipsychotics

The role of atypical antipsychotics in influencing oxidative stress is also matter of discussion [64]. Some authors reported that changes in antioxidant enzymes concentration and activity, together with other biomarkers of oxidative damage, may be independent of antipsychotic treatment and may otherwise represent the results of the pathophysiological process of the disease in patients [22,65]. In addition to this, and before any other consideration, it must be pointed out that redox behavior and performances of any organic compound depends on several parameters, thus a direct comparison is not always possible. In particular, the results from in vitro and in vivo tests may differ due to a number of reaction conditions. Moreover, as pointed out by Janaszewska and Bartosz, even in the context of a simple and preliminary in vitro test, the antioxidant activity of a given compound may appear different when estimated with different tests, due to peculiar indicators or reaction kinetics [57,66]. It must also be stressed that such antioxidant effect could be direct or indirect (mediated by enzymes or other biochemical pathways). Thus, the antioxidant activity should be tested in several models to better evaluate different possible mechanisms and pathways [53]. Several reports suggested that atypical antipsychotics may improve oxidative status, decreasing damage markers [67,68]. Although the mechanism of action is not completely clear, this effect could be exerted by interfering with antioxidant enzymes or by contrasting O_2_^•−^ and hydroxyl radical formation [67]. Other reports indicated that atypical antipsychotics act indirectly by increasing the concentration of the serotonergic metabolite 5-hydroxyindol acetic acid, an efficient scavenger of hydroxyl and superoxide radicals that also contrasts lipid peroxidation [69]. Moreover, Sadowska-Bartosz et al. stressed the relevance of the “local antioxidant action” of atypical antipsychotics, due to their higher local concentration in proximity to dopamine and serotonin receptors. This behavior would result in a protective effect against oxidation, nitration and chlorination of receptors themselves, thus allowing correct receptor functioning and signaling [57].

In rodent models, with the exception of olanzapine, treatment with atypical antipsychotics did not induce significant changes in lipid peroxidation levels, which were also detected after 90 days of treatment. Moreover, previous studies demonstrated that olanzapine and other antipsychotics could stimulate the ROS production, glutathione depletion and lipid peroxidation [70,71]. There is also evidence showing that olanzapine may exert antioxidant activity by upregulating SOD [72,73]. Concerning in vivo effects, a general increase in serum total antioxidant status was observed after 2 months of olanzapine treatment, paralleled by a decrease in serum malondialdehyde levels [74]. More recently, Sadowska-Bartosz et al. presented a study focused on the evaluation of the antioxidant properties of atypical antipsychotics in cell-free and cellular systems. Olanzapine and clozapine were identified as the most efficient antioxidants on the basis of a set of tests investigating the effects of such drugs at the molecular level (DHR123 oxidation, ABTS, DPPH, FRAP, fluorescein bleaching), in agreement with previous observations [53]. The authors rationalized these results by discussing the structural features of the two compounds. In fact, the molecules bear similar functional groups, consisting of a nitrogen-containing moiety behaving as *Lewis* bases capable of donating electrons, thus stabilizing radical species [57]. Clozapine was also observed to be effective in the DPPH radical scavenging test and as a H_2_O_2_ inactivator in a previous study [75,76]. These results are further supported by clinical data, such as the observation of the effects of olanzapine and clozapine in patients, where a decrease in radical-induced damage and neurological symptoms was observed after administration [77]. Moreover, olanzapine is thought to improve SOD functioning [68]. On the other hand, it must be considered that a previous study on schizophrenic patients highlighted that clozapine may induce oxidative stress and pro-apoptotic gene expression in neutrophils [78].

Brinholi et al. investigated the antioxidant properties of atypical antipsychotics in vitro, in comparison with haloperidol. In particular, reactive oxygen species production by neutrophils was measured, and this experiment was paralleled by free radical scavenging assays (DPPH, ABTS) [53]. According to the results of this study, clozapine, olanzapine and risperidone decreased the production of reactive oxygen species. Clozapine, olanzapine and to some extents also ziprasidone, were also reported as good scavengers of the superoxide anion [53]. In agreement with Sadowska-Bartosz et al., [57] Brinholi et al. discussed the results of the study by correlating antioxidant activity and chemical structure, stating that “*the structure and chemical reactivity of the evaluated antipsychotic drugs may explain their antioxidant activity*”. Particularly, the authors focused their attention on the role of the amino group, which is a common feature of such molecules. Thanks to the presence of such a moiety, these molecules behave as *Lewis* bases with an electron donor effect. In addition, clozapine and olanzapine, in particular, bear alkyl groups that facilitate electron donation. This feature may explain the results of the ABTS test. Similarly, as observed in the DPPH assay, oxygen species may be scavenged by the hydrogen donated from the amino group, helped by the resonance of the aromatic ring. In ziprasidone and risperidone, on the other hand, the nitrogen atom is connected through a linker to heterocyclic rings, competing for electronic effects. Moreover, steric effects limit the availability of the nitrogen electron pair. In a similar fashion, the nitrogen atom in quetiapine does not share its electrons easily, due to the presence of oxygen atoms in the ether and hydroxyl groups [53,79]. These observations are, to some extent, supported by previous experiment by Dietrich-Muszalska et al., in which ziprasidone incubation induced more oxidative stress and lipid peroxidation in samples of plasma from healthy subjects than other antipsychotic drugs [71]. More recently, Cai et al. presented a metabolic profiling study aiming at the identification of metabolic pathways affected by oxidative stress and that are responsive to atypical antipsychotic drugs. The authors considered several biomarkers (creatine, choline, inosine, hypoxanthine, uric acid, allantoic acid, lysophosphatidylcholines, phosphatidylethanolamines, corticosterone and progesterone) that were quantified by UPLC-MS/MS in different brain areas of rats. The study highlighted that oxidative damage can interfere with the creatine-phosphocreatine circuit and the purine pathway, leading to increased membrane lipid peroxidation. Treatment with atypical antipsychotics (particularly, clozapine and risperidone) partially restored such impairments by increasing the concentration of creatine, progesterone and phosphatidylethanolamines [33].

Finally, Dietrich-Muszalska et al. reported that atypical antipsychotics may induce different effects on TBARS levels in plasma (indicator of lipid peroxidation) depending on drug dose. Overall, ziprasidone induced pro-oxidant effects in this test, while olanzapine and quetiapine decreased TBARS levels [71].

### 2.3. Aripiprazole

**Scheme 2:**
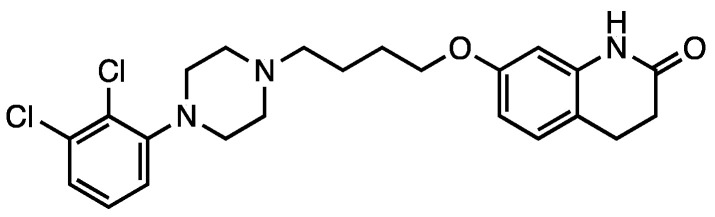
Aripiprazole (**7**).

In the context of antipsychotic treatments, aripiprazole (**7**, Scheme 2) shows a different mechanism of action and, consequently, is referred to as a third-generation agent. This drug acts as a partial agonist on D_2_, D_3_, and 5-HT_1A_ receptors, while is an antagonist for 5-HT_2A_ receptors. Aripiprazole is the first partial dopamine agonist marketed as an antipsychotic drug, and it is also defined as a dopamine-serotonin system stabilizer [52,71]. It is very effective in treating affective, cognitive and negative symptoms of schizophrenia [80].

Park et al. reported that aripiprazole, as well as olanzapine and ziprasidone, could provide protection against oxidative stress in a N-methyl-4-phenylpyridinium (MPP^+^) ion-induced rodent model by modulating ROS levels and SOD activity) and BCL2-associated X protein (Bax) expression [81]. Kato et al. reported that aripiprazole may also contrast microglial O_2_^•−^ generation by interfering with the cascade of protein kinase C (PKC) activation, intracellular Ca^2+^ signaling and NADPH oxidase activation [82].

Aripiprazole was also considered by Cai et al. in the study investigating the therapeutic efficacy of antipsychotics in targeting stress-related metabolic pathways mentioned above. This drug, as well as clozapine and risperidone, was found to be effective in regulating creatine levels in prefrontal cortex and hyppocampus [33].

Dietrich-Muszalska et al. compared the in vitro antioxidant effect of aripiprazole with that of other antipsychotic drugs (haloperidol, clozapine, risperidone, olanzapine, quetiapine and ziprasidone) at concentrations corresponding to their clinically effective doses in the plasma of patients. The effect of such treatment was evaluated by measuring TBARS levels, which is an indicator of lipid peroxidation in plasma. According to the findings of these authors, aripiprazole induced insignificant lipid peroxidation in plasma, whereas it showed antioxidant effects on TBARS level in plasma at higher doses [71].

### 2.4. Other Agents against Oxidative Stress: Natural and Dietary Compounds

**Scheme 3:**
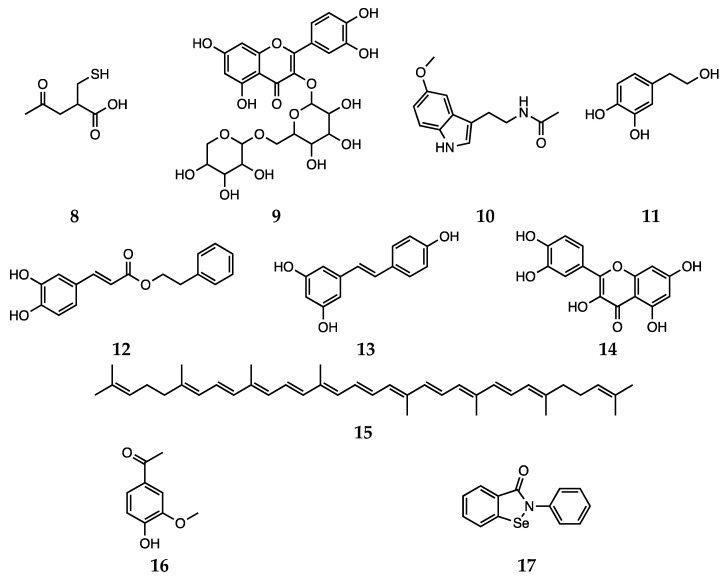
N-acetyl cysteine (**8**), rutin (**9**), melatonin (**10**), hydroxytyrosol (**11**), caffeic acid phenethyl ester (**12**), resveratrol (**13**), quercetin (**14**), lycopene (**15**), apocynin (**16**) and ebselen (**17**).

Besides synthetic antipsychotics, other natural and dietary small molecules have been reported to play an antioxidant role and inactivate harmful reactive species in the context of schizophrenia. The administration of PUFAs to rats represents an explicative example, since an increase in SOD activity was observed [35,83]. The potential of vitamin C (water soluble) and vitamin E (lipid soluble) as antioxidant supplement in patients with schizophrenia was also investigated. However, the use of vitamins C and E does not appear to be a feasible strategy, since the high required dietary intake would most likely result in a pro-oxidant action [84]. Bošković et al. reviewed the contributions reporting studies performed using other supplements, such as N-acetyl cysteine (**8**), rutin (**9**), *Ginkgo biloba*, melatonin (**10**), hydroxytyrosol (**11**), caffeic acid phenethyl ester (**12**), resveratrol (**13**), quercetin (**14**) and lycopene (**15**, Scheme 3) [22]. Various preclinical and clinical studies have shown the positive effects of *Ginkgo biloba* in enhancing cognitive abilities in impaired individuals and reducing anxiety under pathological conditions [85]. Unluckily, due to data heterogeneity and uncertain mechanisms of action, the correct interpretation of such effects is not trivial.

Schiavone and Trabace recently reviewed the scientific contributions from the 2007–2017 timeframe, investigating the effects of antioxidant compounds on neuropathological alterations in psychotic rodent models [86]. *N*-acetylcysteine is among the most studied supplements and showed positive effects in preventing or ameliorating neuropathological and behavioral alterations in the ketamine perinatal model of psychosis and in several other preclinical studies. In particular, this compound induces a normalization of neuropathological alterations in GCLM-KO mice and prevents the negative effects of oxidative stress in CNS postnatal development [87,88,89]. A similar beneficial behavior was also observed for apocynin, a natural compound also known as acetovanillone. A more limited number of contributions also investigated the role of vitamin C, omega-3 fatty acids, and ebselen [86]. In particular, Cabungcal et al. studied the effects of the adolescent treatment with the latter compound, a well-known GPX-mimic, in rats with a neonatal ventral hippocampal lesion, a developmental rodent model of schizophrenia. The authors observed the reversion of behavioral deficits in this animal model, suggesting that pre-symptomatic oxidative stress may represent a potential target for early intervention [90].

## 3. Antidepressant Drugs

### 3.1. Conventional Antidepressants

**Scheme 4:**
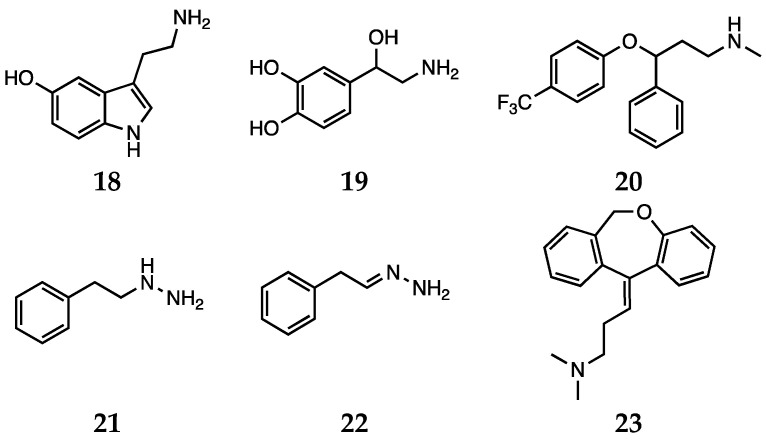
Serotonin (**18**), noradrenaline (**19**), fluoxetine (**20**), phenelzine (**21**), β-phenylethylidenehydrazine (**22**) and doxepin (**23**).

Oxidative stress, as well as proinflammatory signaling, is currently among the most studied causes involved in the pathogenesis of depressive disorders, to the extent that the definition of “oxidative stress hypothesis” is emerging in the context of the onset of depression [91]. In this connection, while monoaminergic-targeted drugs are among the main therapeutic options, other targets such as the glutamatergic system are emerging [27]. As anticipated in the previous sections, in the case of antidepressants the results in ameliorating oxidative stress are also debated [92]. This may be due to the fact that the class of antidepressants is wide and variegated, comprehending different molecules acting through several mechanisms of action [27]. Depression is a multifaceted disease; the neurobiology and molecular events leading to this pathology are still rather unclear. In this connection, monoaminergic-targeted drugs lead the group of clinically available antidepressants, but also the glutamatergic system represents a druggable target [93]. Nevertheless, growing evidence suggests that dietary or commonly administered antioxidants may exert their antidepressant activity by increasing the availability of serotonin (**18**) and noradrenaline (**19**, Scheme 4) in the synaptic cleft, thus acting similarly to the conventional antidepressants [94].

In particular, serotonin and its balance have been extensively studied from this perspective. The selective serotonin reuptake inhibitor (SSRI) fluoxetine (**20**, Scheme 4) decreases oxidative stress levels and improves antioxidant enzyme capacity in rat models [95]. In animal models, chronic administration of the same compound decreased oxidative damage in the cerebral cortex and hippocampus [96]. Moreover, it has been demonstrated that besides direct or indirect antioxidant activity, fluoxetine-induced neuroprotection may result from a mechanism involving serotonin receptor and interference with Nrf2 signaling [97].

Phenelzine (**21**, Scheme 4) (β-phenylethylhydrazine) is a non-selective irreversible MAO inhibitor endowed with antidepressant activity and that has also been reported to be effective in treatment of panic and social anxiety disorder [98,99]. Besides its application in the field of mood disorders, this agent has also been studied for its role of attenuating the effects of oxidative stress in CNS [100,101]. Additionally, in the case of phenelzine, the compound is thought to act through a combination of different mechanisms to contrast oxidative stress (reviewed by Bater et al.) [98]. Phenelzine inhibits γ-aminobutyric acid (GABA) transaminase, thus improving brain GABA levels, and MAOs, that as seen above have a role in contributing to an impaired redox balance. Moreover, the hydrazine group present in the molecule can covalently react with reactive aldehydes, such as acrolein and 4-hydroxy-2-nonenal, which are implicated in oxidative stress and neurodegenerative disorders [98]. Phenelzine is also a MAO substrate, and its interaction with the enzyme leads to the formation of the β-phenylethylidenehydrazine (**22**, Scheme 4) metabolite. This compound is still a weak MAO inhibitor, but more importantly it can markedly inhibit GABA transaminase and primary amine oxidase. Moreover, β-phenylethylidenehydrazine can still sequester aldehydes. As a result, this metabolite can also ameliorate oxidative stress by reducing the presence of byproducts from MAO activity and contrasting the action of hazardous reactive species [98,102,103]. Moreover, phenelzine reduced cell death induced by H_2_O_2_, likely through the scavenging action on hydroxyl radicals [104]. Baker et al. also reported that phenelzine contrasts the effects of the MPP^+^ toxin on differentiated PC12 cells and oxidative damage induced by peroxynitrite in plasma lipids and proteins [98].

Doxepin (**23**, Scheme 4), which is commonly used to treat major depression, was proved to be a potent antioxidant through a combination of in vitro tests. In particular, Palchoudhuri and colleagues reported the positive results obtained with doxepin in a phosphomolybdenum assay and detected ferric and cupric ions reducing activity, ferrous ion chelating properties, hydrogen peroxide and nitric oxide scavenging activity [105].

### 3.2. Natural Compounds

**Scheme 5:**
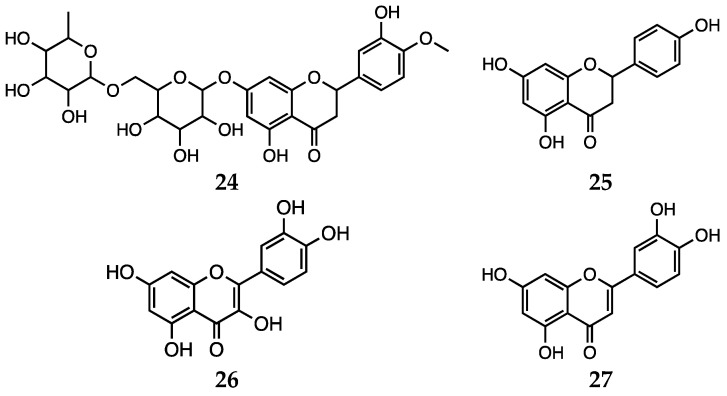
Hesperidin (**24**), naringenin (**25**), quercetin (**26**) and luteolin (**27**).

Herbal preparations are attracting growing interest, since therapies based on the use of natural compounds represent promising alternatives or supporting remedies for psychiatric disorders, including depression [44,106,107]. In fact, natural substances are generally well perceived by patients, since they are thought to possess better positive effects with less toxicity. Hritcu et al. recently reviewed the mechanisms by which antidepressant flavonoids exert antioxidant activity [43]. Concerning the first pharmacological activity, several pathways influenced by flavonoids are thought to play a role. The compounds from this class act by preventing mitochondrial membrane potential dissipation, modulating intracellular Ca^2+^ concentration and K^+^ channels, downregulating Bax, caspases 3, caspase 9, cytochrome C but upregulating Bcl expression and by interfering with ERK and AKT pathways. More interestingly, as anticipated, some flavonoids were reported to strengthen the pharmacokinetic efficacy of canonical medications for depression. In particular, hesperidin (**24**) and naringenin (**25**, Scheme 5) enhanced the area under the curve (AUC), maximum plasma concentration (*C*_max_), and elimination half-life (*t*_1/2_) of rasagiline (MAO-B inhibitor). Moreover, quercetin (**26**, Scheme 5) was effective in interfering with glutamatergic neurotransmission in rodent models [108,109]. On the other hand, with respect to the antioxidant role, flavonoids generally exert their effects through a combination of the following: alteration of cytokine levels, influence on oxidative stress and tuning of energy metabolism parameters [43,110,111]. In this case, the observed antioxidant effect may be also considered as the result of concerted processes, that can be resumed in the combination of direct (quenching free radicals) and indirect (stimulation of antioxidant enzymes) mechanisms [43]. Concerning the indirect mechanisms, in general, flavonoids are reported to enhance SOD and GSH levels, as well as glutathione reductase (GR) activity. Meanwhile, other compounds such as luteolin (**27**, Scheme 5) and quercetin (**26**) inhibit free radical generating enzymes, such as myeloperoxidase, xanthine oxidase, lipoxygenase, microsomal monooxygenase, and NADPH oxidase. Another reported antioxidant mechanism consists of the ability of binding or chelating metal ions (iron, copper, zinc), thus making them less accessible to oxidation and showing antiradical properties [112,113,114].

*N*-acetylcysteine has been nominated as a possible new therapeutic agent to be combined with psychopharmacotherapy, especially in the context of depressive disorders. It modulates the activity of the glutamate transporter, and it is also a chelating agent for heavy metals, it reduces inflammatory markers, it is a protective agent against the effects of mitochondrial dysfunctions, and it inhibits apoptosis and promotes neurogenesis and neural survival [115,116].

Concerning PUFAs, tests on stem cells converted into nervous cells showed that fish oil in combination with psychotropic drugs could be an effective treatment for depression. It has also been noted that the action of fish oil was mainly carried out in glial cells, emphasizing the important role of these cells in the pathogenesis of depression [117].

## 4. In Silico Approaches

The overview thus far gives a quite good picture of how already existing compounds can have tandem beneficial effects in treating major mental disorders and reducing oxidative stress levels. The employment of machine learning algorithms developed in the last few years to the possible use of antipsychotic or antidepressant drugs as effective antioxidants has also seen a decisive contribution of many researchers [118,119,120,121,122,123,124,125]. However, many of the studies performed so far made use of statistical or classical mechanics-based methodologies (like QSAR, molecular docking and molecular dynamics) [126,127,128]. These approaches, while being very useful when dealing with a large number of trial molecules, do not have the ability to elucidate the intrinsic molecular mechanism underlying the efficacy of a particular structure. For this task, one needs resort to methods based on quantum mechanical (QM) calculations.

Density functional theory (DFT) is nowadays one of the most applied QM methods, since it can be utilized on fairly large structures without many trade-offs with respect to other more accurate methods, which require a much larger computational cost. Although, when considering the mechanism of action of drugs, one is faced by the very complicated structure of human physiology, the ability of knowing, at a molecular level, the intrinsic mechanisms giving rise to an effective compound is a powerful tool. For example, it paves the way to the employment of rational design to improve already existing structures or propose new ones with a potentially higher activity still.

**Scheme 6:**
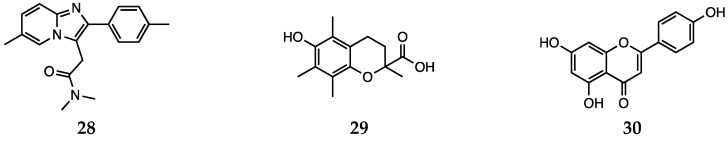
Zolpidem (**28**), Trolox (**29**), and apigenin (**30**).

While DFT studies on antipsychotic drugs [129,130,131,132] and of antidepressants [133,134,135] are commonly found in the literature, works on the antioxidant activity of such compounds are still scarce. In a recent study by some of us, [123] the free radical scavenging activity of fluoxetine (20) and serotonin (**18**, Scheme 4) was investigated using a meta-hybrid functional (M06-2X [136]) in the gas phase and in the solvent. The study confirmed the notion that although fluoxetine possesses some radical scavenging capacity on its own, it is less active than serotonin itself. Thus, the effect it exerts as an oxidative stress balancer most likely comes from the higher concentration of free serotonin found when the drug is taken. In addition, the employment of DFT computations allowed the authors to analyze the antioxidant activity of each available site for a range of different mechanisms, giving a complete picture of the overall mechanism of antioxidant activity of fluoxetine and serotonin. Another work by some of the authors of this review instead considered another compound, namely zolpidem (**28**, Scheme 6), a drug usually administered to fight insomnia [124]. The antioxidant ability of this molecule via the hydrogen atom transfer (HAT) mechanism was investigated at the SMD-M06-2X/6-311 + G(d,p)//M06-2X/6-31G(d) level of theory and was found to be comparable to that of melatonin (**10**, Scheme 3) or Trolox (**29**, Scheme 6) (an analog of vitamin E). In addition, in this case, all the sites capable of having any activity towards free radicals were tested and the most reactive were singled out and analyzed on the basis of their chemical nature. In particular, it emerged that fluoxetine, zolpidem and serotonin, in analogy to melatonin, possess a reactive CH_2_ site from which HAT is efficient towards alkoxyl as well as peroxyl radicals. These two studies open promising routes as the peculiar motifs giving fluoxetine, serotonin and zolpidem their antioxidant activity, and can be regarded as a predictive factor for antioxidant activity, and this can be extended also to other drugs to assess their antioxidant capacity.

Conversely, the antioxidant properties of dietary and natural compounds that can have beneficial effects in the treatment of psychological or behavioral disorders have been extensively studied. In particular, a model for the assessment of total antioxidant capacity in solution has been recently proposed and successfully applied to molecules such as melatonin and Trolox by Galano et al. [137]. In addition, the same group studied the antioxidant properties of melatonin and *N*-acetylcysteine amide in depth [138,139].

Melatonin was investigated with a meta-hybrid functional (M05-2X [140]) in benzene and water using a continuum solvation approach (IEFPCM [141,142,143]) and its possible reaction mechanisms were evaluated and ranked. It was found that it is an excellent ^•^OH scavenger and a very good quencher of reactive peroxyl radicals (such as ^•^OOCCl_3_) that in aqueous solution reacts mainly through a sequential electron proton transfer (SEPT) mechanism. Moreover, in a later work, possible derivatives of melatonin were investigated computationally for enhanced antioxidant capabilities [144]. The results, obtained again with the M05-2X functional, predict five of the investigated derivatives to have a higher antioxidant activity than melatonin.

The amide of *N*-acetylcysteine (**8**, Scheme 3) was also the topic of a DFT computational study employing the M05-2X functional [139]. The results show a diffusion-controlled reaction in both aqueous and apolar media for the reaction with ^•^OH with the same mechanism for the two environments. However, in the polar solution, the S atom was seen to be the most reactive site, whereas in benzene, the S atom and the C atom directly bound to it equally contributed to the antioxidant activity. These findings were confirmed by another successive study that highlighted the preference for the HAT mechanism for the sulfhydryl group present in five investigated drugs, among which *N*-acetylcysteine [145]. A notable aspect of this work is that *N*-acetylcysteine was ranked lowest among the five tested molecules.

The beneficial properties of naringenin (**25**, Scheme 5) in limiting oxidative stress were also considered in a recent computational study [146]. Computations carried out at the M06-2X/6-311+ G** level of theory gave a good picture of the effects exerted by electron-withdrawing and electron-donating groups on the antioxidant activity of this compound in the gas phase, in benzene and in water. Moreover, different scavenging mechanisms were analyzed and it emerged that in the gas phase and in benzene, HAT is the most favorite mechanism, whereas in water, a sequential proton loss electron transfer (SPLET) occurs, confirming the results obtained in a previous computational work [147].

Luteolin (**27**, Scheme 5) and apigenin (**30**, Scheme 6) were screened in a combined experimental and computational study to assess the effect of the complexation of Cu(II) to two very similar flavonoids [148]. Authors found an opposite effect on the antioxidant ability upon the insertion of a copper cation. In the case of luteolin, an increased activity was revealed, whereas apigenin showed a lowered antioxidant capability. The role of copper in the enhanced reactivity of luteolin was found to be directly connected with the lower ionization potentials of the Cu-luteolin complexes with respect to the sole luteolin molecule.

The literature on the antioxidant properties of flavonoids in natural compound is extensive. In this section, a brief overview limited to the results obtained on compounds that have shown possible application in the treatment of schizophrenia or depression was presented. For a more exhaustive and general treatment of the subject, a number of recent reviews have been published [149,150,151,152].

This analysis shows how a very rich literature regarding the general antioxidant activity of flavonoids is paralleled by a very scarce collection of works that investigate the antioxidant properties of antidepressant and antipsychotic drugs at a molecular level. One of the few very recent contributions in this field, reported by some of the authors of this work, originated from the idea to combine the antioxidant abilities of fluoxetine and of selenium [153]. Although an oligoelement in the human body, selenium is found in some essential antioxidant enzymes such as glutathione peroxidases, iodothyronine deiodinase (DIO), thioredoxin reductases (TrxR) and methionine sulfoxide reductases (Msr) [154]. Among those, GPx has a central role in balancing cellular oxidative stress, and its mechanism of action [155,156,157,158] has inspired the design and testing of many biomimetic compounds targeted at the prevention of oxidative damage [159,160,161]. Many possible different structures and model chemical motifs have been computationally evaluated to be the starting scaffold on which to construct an efficient and effective antioxidant drug [162,163,164,165]. The good results obtained in those studies lay out some very promising perspectives for the combination of the intrinsic antioxidant properties of selenium with ad hoc designed molecules to comprise multiple target abilities in a single compound.

## 5. Conclusions

In this review, we have discussed the antioxidant potential of several antipsychotic and antidepressant drugs, which has been reported in numerous cases in literature, and constitutes a valuable additional potential of these molecules. These results are often controversial, and a clear rationale and a common thread is still missing. Undoubtedly, this is due both to the intrinsic complexity of the physiological environment and the difficulty of detection of meaningful biomarkers. Nevertheless, as complementary to clinical observation and to in vitro and in vivo studies, we emphasize the importance of in silico approaches, aimed at assessing the antioxidant potential based on the molecular structure and identify peculiar chemical motifs which can be exploited for a rational drug design. Since oxidative stress has been clearly linked to the onset and development of mental disorders, new research on the molecular bases of the action of active antipsychotic and antidepressant drugs is needed to better understand the causes underlying their efficacy in their psychotropic action and their potential novel task as antioxidants.

## List of Acronyms

Central nervous system	CNS
Reactive oxygen species	ROS
Reactive nitrogen species	RNS
Deoxyribonucleic acid DNA	DNA
Polyunsaturated fatty acids	PUFAs
Superoxide dismutase	SOD
Glutathione peroxidase	GPx
Catalase	CAT
Glutathione	GSH
Thiobarbituric acid related substances	TBARS
Monoamine oxidase A	MAO-A
Monoamine oxidase B	MAO-B
Dopamine	DA
Catechol-O-methyl transferase	COMT
6-hydroxydopamine	6-OHDA
8-hydroxy-2′-deoxyguanosine	8-OHdG
Dihydrorhodamine 123	DHR123
2,2′-azinobis(3-ethylbenzthiazoline-6-sulfonate)	ABTS
2,2-diphenyl-1-picryl-hydrazyl-hydrate	DPPH
Ferric reducing antioxidant power	FRAP
Ultra-performance liquid chromatography-tandem mass spectrometry	UPLC-MS/MS
N-methyl-4-phenylpyridinium	MPP^+^
Nicotinamide adenine dinucleotide phosphate	NADPH
Glutamate-cysteine ligase modifier subunit knockout	GCLM-KO
Γ-aminobutyric acid	GABA
Extracellular-signal-regulated kinase	ERK
Protein kinase B	AKT
area under the curve	AUC
Quantitative structure activity relationship	QSAR
Quantum mechanics	QM
Density functional theory	DFT
Hydrogen atom transfer	HAT
Integral equation formalism polarizable continuum model	IEFPCM
Sequential electron proton transfer	SEPT
Sequential proton loss electron transfer	SPLET
Iodothyronine deiodinase	DIO
Thioredoxin reductases	TrxR
Methionine sulfoxide reductases	Msr
